# Impact of c-di-GMP on the Extracellular Proteome of *Rhizobium etli*

**DOI:** 10.3390/biology12010044

**Published:** 2022-12-26

**Authors:** María J. Lorite, Ariana Casas-Román, Lourdes Girard, Sergio Encarnación, Natalia Díaz-Garrido, Josefa Badía, Laura Baldomá, Daniel Pérez-Mendoza, Juan Sanjuán

**Affiliations:** 1 Department of Soil and Plant Microbiology, Estación Experimental del Zaidín, CSIC, 18008 Granada, Spain; 2 Centro de Ciencias Genómicas (CCG), Universidad Nacional Autónoma de México (UNAM), Cuernavaca 62210, Morelos, Mexico; 3 Secció de Bioquímica i Biología Molecular, Departament de Bioquímica i Fisiologia, Facultat de Farmàcia i Ciències de l’Alimentació, Universitat de Barcelona, 08028 Barcelona, Spain; 4 Institut de Biomedicina de la Universitat de Barcelona (IBUB), Institut de Recerca Sant Joan de Déu (IRSJD), 08028 Barcelona, Spain

**Keywords:** cyclic diguanylate, rhizobia, protein PTM, extracellular proteins, moonlighting proteins, adhesins

## Abstract

**Simple Summary:**

The second messenger cyclic diguanylate (c-di-GMP, cdG) is a bacterial lifestyle-switch molecule, well known for its role in biofilm formation. Extracellular biofilm matrix components include diverse biopolymers such as polysaccharides, nucleic acids, proteins and lipids. The production and/or secretion of many these matrix biopolymers can be directly or indirectly regulated by cdG. We studied the extracellular proteome of a *Rhizobium etli* strain expressing artificially high levels of intracellular cdG. We found that, in addition to promoting the secretion of various extracellular proteins likely involved in adhesion and biofilm formation, high cdG levels also promote the export of cytoplasmic proteins (ECP) to the cell exterior. Intriguingly, most these cytoplasmic proteins have been previously described as moonlighting or multifunctional proteins in other organisms, often found extracellularly or at the bacterial cell surface. We obtained evidence that this ECP may involve an active process that would be enhanced by cdG. For a typical cytoplasmic protein, glyceraldehyde 3-phosphate dehydrogenase (Gap), we also observed that cdG increases the number of extracellular Gap proteoforms that can be separated by two-dimensional gel electrophoresis. The results suggest that cdG promotes the active exportation of cytoplasmic proteins through yet unknown mechanisms involving the post-translational modification of proteins.

**Abstract:**

Extracellular matrix components of bacterial biofilms include biopolymers such as polysaccharides, nucleic acids and proteins. Similar to polysaccharides, the secretion of adhesins and other matrix proteins can be regulated by the second messenger cyclic diguanylate (cdG). We have performed quantitative proteomics to determine the extracellular protein contents of a *Rhizobium etli* strain expressing high cdG intracellular levels. cdG promoted the exportation of proteins that likely participate in adhesion and biofilm formation: the rhizobial adhesion protein RapA and two previously undescribed likely adhesins, along with flagellins. Unexpectedly, cdG also promoted the selective exportation of cytoplasmic proteins. Nearly 50% of these cytoplasmic proteins have been previously described as moonlighting or candidate moonlighting proteins in other organisms, often found extracellularly. Western blot assays confirmed cdG-promoted export of two of these cytoplasmic proteins, the translation elongation factor (EF-Tu) and glyceraldehyde 3-phosphate dehydrogenase (Gap). Transmission Electron Microscopy immunolabeling located the Gap protein in the cytoplasm but was also associated with cell membranes and extracellularly, indicative of an active process of exportation that would be enhanced by cdG. We also obtained evidence that cdG increases the number of extracellular Gap proteoforms, suggesting a link between cdG, the post-translational modification and the export of cytoplasmic proteins.

## 1. Introduction

The second messenger bis-(3′,5′)-cyclic diguanosine monophosphate (cyclic diguanylate, c-di-GMP, cdG) is a bacterial lifestyle-switch molecule, with a crucial role in the transition from a planktonic/motile to a sessile biofilm mode of growth, but also with a great influence on various key cellular processes, including cell–cell signalling, cell cycle progression and virulence [1,2]. Cyclic-di-GMP signalling systems are generally composed of four major constituents: (i) diguanylate cyclases (DGCs, synthesize cdG from two GTP molecules), (ii) phosphodiesterases (PDEs, degrade cdG), (iii) cdG binding effectors that interact with (iv) target components to produce a molecular output [3,4]. The complexity of cdG regulation, compared with other well-known nucleotide second messengers such as cAMP, comes from the multiplicity of cdG metabolising (synthesis and degradation) enzymes, the wide variety of cdG effectors (diverse proteins, transcriptional regulators, RNA motifs) and the multitude of molecular targets so far described [1,5]. Indeed, regulation based on cdG signalling can take place at transcriptional, posttranscriptional and posttranslational levels.

As mentioned above, cdG is best known for its involvement in the bacterial decision to attach to a surface and form a biofilm community. The biofilm is a dynamic tridimensional structure where bacteria live encased in a self-produced extracellular matrix, whose structural and functional properties are essential for social cooperation, resource capture and enhanced survival [6]. Extracellular matrix components include diverse biopolymers such as polysaccharides, nucleic acids, proteins and lipids. The production and/or secretion of many these matrix biopolymers are known to be directly or indirectly regulated by cdG. For instance, many cdG-regulated exopolysaccharides have been described (reviewed by Pérez-Mendoza and Sanjuán [7]). Exopolysaccharides are a major fraction of the biofilm matrix and often essential for biofilm formation, since they can protect bacteria from desiccation, enhance survival in stressful environments, mediate cell adhesion and modulate plant and animal infections when interacting with eukaryotic hosts.

Proteins are important components of bacterial biofilm matrices essential for maintaining structural integrity [8], including actively secreted proteins, adhesins and motility organelles (flagella, pili), many of them important for biofilm structural properties. Similar to polysaccharides, the production or secretion of certain adhesins and other matrix proteins can be directly or indirectly regulated by cdG [9,10]. CdG can also affect the expression or activity of several protein secretion systems [9,11,12,13,14]. Moreover, cytoplasmic proteins have also been found in bacterial extracellular matrices [8,15,16,17], either in single strain laboratory biofilms (e.g., [17]) and in naturally occurring, complex biofilms [16]. Although the presence of cytoplasmic proteins in extracellular compartments has been presumed to originate from cell lysis [18] or to be part of the cargo in outer membrane vesicles (OMVs; [17], many authors support that certain cytoplasmic proteins can be secreted outside the cell by yet unknown, so-called non-classical secretion systems [19,20,21,22]. In a few instances, extracellular cytoplasmic proteins have been shown to use known secretion pathways such as a modified T3SS [23].

Despite the significant advances, knowledge on the molecular mechanisms of cdG regulation and the role of this second messenger in bacterial life cycles is still fragmentary and far from complete. This is especially true in plant-interacting bacteria, with a paucity of information on their cdG signal transduction systems, compared to free-living or animal-interacting bacteria [14,24].

The aim of this work was to identify extracellular proteins regulated by c-di-GMP in the legume symbiotic bacterium *Rhizobum etli*. 2D-GE and iTRAQ-labelling quantitative proteomics were used to identify differential protein abundances between two *R. etli* isogenic strains, one of which expresses a constitutive diguanylate cyclase to produce high levels of intracellular c-di-GMP.

## 2. Materials and Methods

### 2.1. Bacterial Strains and Growth Conditions

Strains LR101 and LR102 [25] are derivatives of *Rhizobium etli* CFN42. LR101 carries a mini-Tn7pleD*Km transposon and expresses high levels of intracellular c-di-GMP. LR102 was used as the control strain, and it carries a mini-Tn7Km transposon and expresses wild-type levels of c-di-GMP. Rhizobial strains were routinely grown in tryptone-yeast extract-CaCl_2_ (TY; [26]) at 28 °C. For protein and RNA isolation, bacteria were grown in minimal medium MMY [27], containing succinate and ammonium chloride as C or N sources, respectively. When required, antibiotics nalidixic acid (20 µg/mL) and kanamycin (50 µg/mL) were added.

### 2.2. Protein Sample Preparation

Cells from exponentially growing TY cultures of LR101 and LR102 were used to inoculate 500 mL MMY flasks to an OD_600_ of 0.1. Cultures were grown O/N in a shaker incubator at 28 °C and 180 rpm, to an OD_600_ of 0.6–0.7. Cultures were centrifuged for 2 × 1 h at 7500× *g*. After the second centrifugation, supernatants were carefully collected and immediately frozen at −80 °C. Cell pellets were washed with saline solution, frozen in liquid nitrogen and kept at −20 °C until use.

Supernatants were lyophilized in a freeze-dryer Thermo Savant ModulyoD and then resuspended in 15 mL of extraction buffer [28]. Cell pellets were resuspended in 1 mL of extraction buffer and sonicated for 5 cycles of 30 s (C = 4, 20%) in a Branson Sonicator 250, then centrifuged at 15,000× *g* for 2 min. The phenol extraction protocol [28] was followed for protein isolation. The protein obtained was determined and quantified by a Bradford assay from Bio-Rad [29] and with PierceTM 660 nm Protein Assay from Thermo Scientific (Waltham, MA, USA).

### 2.3. Proteomics

The methods for analytical 2-DE, image analysis, preparative 2-DE gels and run were done essentially as reported previously [30].

After phenol extraction, proteins were diluted in sample buffer containing 7 M urea, 2 M thiourea, 4% (*w*/*v*) 3-[(3-cholamidopropyl)-dimethylammonio]-1-propanesulfonate (CHAPS), 2% ampholytes (1.5% pH range 4–8 and 0.5% pH range 3–10) and 60 mM DTT. The proteins were quantified by the Bradford method and stored at −20 °C until analysis. Two-dimension preparative gel electrophoresis (2D) was performed as previously described by Encarnación et al. [30], using 500 ug per sample. 2D gels were coomassie blue G-250 stained [31], and then were scanned (GS800 Calibrated Imaging Densitometer, Bio-Rad, Hercules, CA, USA) for their analysis by means of the PDQuest 8.0.1 PDQuest software (Bio-Rad Laboratories, Inc., Hercules, CA, USA). Spots that showed a two-fold change from at least three gels for each group, and a significance level of 95% (Student’s *t*-test; *p* ≤ 0.05) were excised and trypsin digested. The peptide mass fingerprint was obtained using a MALDI-TOF Autoflex (Bruker Daltonics). One hundred satisfactory shots and contaminants were not excluded. The spectrum was annotated by the FlexAnalysis 1.2 v SD1 Patch 2 (Bruker Daltonics). The search engine MASCOT server 2.0 was used to compare the fingerprints against the *Rhizobium etli* database release 2012 with the following parameters: one missed cleavage allowed, carbamidomethyl cysteine as the fixed modification and oxidation of methionine as the variable modification. We accepted those proteins with scores greater than 50 and a *p* < 0.05.

Quantitative proteomics were carried out at the CNB-CSIC Proteomics Facility (Madrid, Spain; http://proteo.cnb.csic.es/proteomica/; (accessed on 22 November 2022). Protein samples were subjected to S-Trap^TM^ digestion followed by tagging with iTRAQ 4plex^TM^ reagent. Protein digestion in the S-Trap filter (Protifi, Huntington, NY, USA) was performed following the protocol described in Ciordia et al. [32], with slight modifications. Briefly, 50 µg of protein of each sample was diluted to 40 µL with 5% SDS. Afterwards, 12% phosphoric acid and then seven volumes of binding buffer (90% methanol; 100 mM TEAB) were added to the sample (final phosphoric acid concentration: 1.2%). After mixing, the protein solution was loaded to an S-Trap filter in two consecutive steps, separated by a 2 min centrifugation at 3000× *g*. Then, the filter was washed 3 times with 150 μL of binding buffer. Finally, 1 µg of Pierce MS-grade trypsin (Thermo-Fisher Scientific, Waltham, MA, USA) in 20 μL of a 100 mM TEAB solution was added to each sample in a ratio 1:20 (*w*/*w*) and spun through the S-Trap prior to digestion. Flow-through was then reloaded to the top of the S-Trap column and allowed to digest in a wet chamber at 37 °C overnight. To elute peptides, two step-wise buffers were applied: (1) 40 μL of 25 mM TEAB and (2) 40 μL of 80% acetonitrile and 0.2% formic acid in H_2_O, separated by a 2 min centrifugation at 3000× *g* in each case. Eluted peptides were pooled and vacuum centrifuged to dryness.

The resulting peptides were subsequently labelled using the iTRAQ-4plex Isobaric Mass Tagging Kit (SCIEX, Foster City, CA, USA) according to the manufacturer’s instructions. After labelling, the samples were pooled, evaporated to dryness and stored at −20 °C until the LC−MS analysis. Four biological replicates of each condition were analysed in two different experiments.

Peptide concentration was carried out by Qubit™ Fluorometric Quantitation (Thermo Fisher Scientific, Waltham, MA, USA). A 1 µg aliquot of each iTRAQ experiment was subjected to 1D-nano LC ESI-MS/MS (Liquid Chromatography Electrospray Ionization Tandem Mass Spectrometric) analysis using an Ultimate 3000 nano HPLC system (Thermo Fisher Scientific, Waltham, MA, USA) coupled online to a Orbitrap Exploris 24 0 equipped with a FAIMS Pro ion source (Thermo Fisher Scientific, Waltham, MA, USA). Peptides were eluted onto a 50 cm × 75 μm Easy-spray PepMap C18 analytical column at 45 °C and were separated at a flow rate of 300 nL/min using a 120 min gradient ranging from 2% to 95% mobile phase B (mobile phase A: 0.1% formic acid (FA); and mobile phase B: 80% acetonitrile (ACN) in 0.1% FA). The loading solvent was 2% ACN) in 0.1% FA and injection volume was 5 µL. Data acquisition was performed using a data-dependent top-20 method, in full scan positive mode, scanning 375 to 1200 m/z. Survey scans were acquired at a resolution of 60,000 at m/z 200, with a Normalized Automatic Gain Control (AGC) target (%) of 300 and a maximum injection time (IT) in AUTO. The top 20 most intense ions from each MS1 scan were selected and fragmented via Higher-energy collisional dissociation (HCD). Resolution for HCD spectra was set to 45,000 at m/z 200, with an AGC target of 100 and a maximum ion injection time in AUTO. Isolation of precursors was performed with a window of 0.7 m/z, exclusion duration (s) of 45 and a HCD collision energy of 30. Precursor ions with single, unassigned or six and higher charge states from fragmentation selection were excluded.

Proteomics data analysis were performed using 4 search engines (Mascot, OMSSA, X!Tandem and Myrimatch) and a target/decoy database built from sequences in the *Rhizobium etli* proteome at Uniprot Knowledgebase. Search engines were configured to match potential peptide candidates with a mass error tolerance of 10 ppm and fragment ion tolerance of 0.02 Da, allowing for up to two missed tryptic cleavage sites and a maximum isotope error (^13^C) of 1, considering fixed carbamidomethyl on cysteine and variable oxidation of methionine, pyroglutamic acid from glutamine or glutamic acid at the peptide N-terminus and modification of lysine and peptide N-terminus with iTRAQ 4-plex reagents. Score distribution models were used to compute peptide-spectrum match *p*-values [33], and spectra recovered by a FDR* <= 0.01 (peptide-level) filter were selected for quantitative analysis. Approximately 5% of the signals with the lowest quality were removed prior to further analysis. Differential regulation was measured using linear models [34] and statistical significance was measured using q-values (FDR, False Discovery Rate). All analyses were conducted using software from Proteobotics (Madrid, Spain).

### 2.4. Western Blots

For 1D-GE Western Blots (WB), protein samples (20–40 µg) were loaded onto 10% SDS-PAGE gels and electrophoresed in Tris-Glycine buffer in a Mini-Protean Tetra Cell from Bio-Rad [35]. Gels were transferred to PVDF membranes using the Trans-Blot Turbo Transfer System from Bio-Rad with the Mixed-MW protocol supplied from the manufacturer. PVDF membranes were activated with methanol, washed with PBS-T buffer (137 mM NaCl, 2.7 mM KCl, 1.76 mM, KH_2_PO_4_, 10 mM Na_2_HPO_4_, 0.1% Tween 20, pH 7.4) and blocked with 2% skimmed milk in PBST. Blocked PVDF membranes were incubated overnight at 4 °C in the same buffer with the primary antibody. After washing, the membrane was incubated with a 1:10,000 dilution of the peroxidase-linked secondary antibody. Detection was done with ECL Prime Western Blotting from Amersham in a Chemidoc XRS System from Bio-Rad. Band analysis was performed with PD Quest or Quantity-One programs supplied by Bio-Rad. Bio-Rad Broad Range Unstained SDS-PAGE Standard from 6.5 to 200 kDa was used as a molecular mass marker.

Two-dimensional electrophoresis protein separation for Western blots were carried out at the university of Córdoba Proteomics facility (https://www.uco.es/investigacion/portal/proteomica; accessed on 22 November 2022). For 2D-GE WB, 80 µg protein samples were used. The first dimension was run in a 7 cm IPG (Immobilized pH gradient) strip pH 3–10 from Bio-Rad, with a lineal increase of voltage (2 h of passive rehydration; 10 h at 50 V of active rehydration; 30 min at 250 V; 1 h at 1000 V; 30 min at 4000 V; and 4000 V until 20,000 Vh). Strips were equilibrated for two periods of 15 min in Tris-Urea buffer (375 mM Tris-HCl, pH 8.8, 6 M Urea, 2% SDS, 20% glycerol), with 2% DTT added the first time and 2.5% of Iodoacetamide added secondly. For the second dimension, the strips were transferred into a vertical 10% SDS-PAGE and run at 50 V in a MiniProtean Tetra Cell System (BioRad, Hercules, CA, USA). Then, 2D SDS-PAGE gels were stained with imidazole and scanned in a GS-800 Calibrated Densitometer (Bio-Rad). Gels were washed for 10 min with 100 mM EDTA and equilibrated for 15 min in a Towbin buffer (25 mM Tris-HCl pH 8.3, 192 mM Glicina, 20% Metanol, 0.02% SDS). Finally, gels were transferred to nitrocellulose membranes for 20 h at 30 V and 2 °C in a Trans-Blot Plus Electrophoretic Transfer Cell (BioRad). Protein immunodectection was carried out as above. Monoclonal mouse anti-E. coli Elongation Factor EF-Tu antibody was diluted 1:2000 times and used following the manufacturer’s instructions (LSBio, Inc., Seattle, WA, USA). This antibody recognizes the N-terminus (SKEKFE sequence) of the EF-Tu from *Escherichia coli* and many other bacteria. Rabbit polyclonal anti-*R. etli* Gap antibodies were raised (Davids Biothecnologie GMBH, Germany) against a synthetic peptide (dlgpvetnahllrydsihgk) derived from the *R. etli* CFN42 Glyceraldehyde 3-phosphate dehydrogenase (Gap; RHE_CH03496) primary sequence. These anti-Gap antibodies were diluted to a working concentration of 10 µg/mL.

### 2.5. Membrane Vesicles Isolation and Quantification

Membrane Vesicles (MVs) were isolated from culture supernatants following previously described methodology [36]. Bacterial cells were grown in MMY cultures with the pertinent antibiotics until an OD_600_ of 0.6–0.8 was reached, then pelleted by centrifugation at 10,000× *g* for 30 min at 4 °C. The supernatants were filtered through a 0.45 μm-pore-size filter (Millipore) and concentrated by centrifugation through a 100 KDa Centricon^®^ Plus-70 filter device (Millipore). The MVs were collected by ultracentrifugation at 100,000× *g* for 2 h at 4 °C, washed with phosphate buffered saline (PBS) to eliminate excess polysaccharide and again pelleted at 100,000× *g* for 2 h at 4 °C. The pellet containing the MVs was resuspended in an appropriate volume of PBS and stored at −20 °C.

MVs were quantified by lipid content, which was determined using the lipophilic fluorescent dye FM4-64 (Thermofisher, Waltham, MA, USA) as previously described [37]. A fraction of PBS resuspended MVs was incubated with FM4-64 (final concentration of 5 μg/mL in PBS) for 5 min in darkness at room temperature. Vesicles alone and the FM4-64 probe alone were used as negative controls. After excitation at 515 nm, emission at 635 nm was measured with the multiplate reader Varioskan TM LUX (ThermoScientific, Waltham, MA, USA). Fluorescence was normalized for the culture volume from which MVs were isolated.

### 2.6. Transmission Electron Microscopy Immunolabeling

Samples were prepared at the Electron Cryomicroscopy Unit from the CCiTUB, University of Barcelona. They were cryoimmobilized using a Leica HPM100 High-Pressure Freezer (Leica Microsystems, Vienna, Austria). Planchettes containing the frozen samples were transferred to cryo-tubes containing 0.5% uranyl acetate (EMS, Hatfield, PA, USA) in acetone under liquid nitrogen and were freeze substituted at −90 °C for 80 h in an EM AFS2 (Leica Microsystems, Vienna, Austria). Samples were warmed up to −50 °C at 5 °C/h slope and kept at −50 °C. They were rinsed with acetone and infiltrated in Lowicryl HM20 resin (EMS, Hatfield, PA, USA) at −50 °C. Samples were polymerized under UV light: at −50 °C for 24 h, during the warming up at 5 °C/h slope until 22 °C and at 22 °C for 48 h. Sections 60 nm in thickness were obtained using a UC6 ultramicrotome (LeicaMicrosystems, Vienna, Austria). They were washed sequentially in 10 mM PBS, glycine 10 mM and 10 mM PBS. Then, they were incubated on drops of 5% bovine serum albumin (BSA) in 10 mM PBS for 15 min and they were changed to 1% BSA in 10 mM drops, followed by incubation with the polyclonal anti-*R. etli* Gap antibody 1:35 in 1% BSA 10 mM PBS for 1 h. Then, they were washed in 0.25% Tween 20 in PBS 10 mM and they were changed to 1% BSA in 10 mM PBS, followed by incubation with anti-rabbit 12 nm colloidal gold-conjugated antibody 1:30 (Jackson) in 1% BSA 10 mM PBS for 30 min. Samples were washed in PBS, incubated in 1% glutaraldehyde in PBS for 5 min and rinsed in milliQ water. As a negative control for non-specific binding of the colloidal gold-conjugated antibody, the primary antibody was omitted. Sections were stained with 2% uranyl acetate and lead citrate and were observed in a Tecnai™ Spirit TWIN microscope (FEI, Eindoven, The Netherlands) equipped with a tungsten cathode from the Electron Cryomicroscopy Unit from the CCiTUB. Images were acquired at 120 kV with a CCD Megaview 1 k × 1 k.

### 2.7. Bacterial Growth Curves and Cell Viability Determination

Growth curves were carried out in standard culture conditions. Pre-cultures of the bacteria were grown in a TY medium supplemented with the required antibiotics at 28 °C to an OD600 = 1. The bacterial pre-cultures were washed and diluted 1/20 in the fresh medium, and 200 µL were dispensed in wells of a polystyrene microplate (Nunc MicroWell 167008). Four replicates for each strain and condition were loaded per plate. The plates were incubated at 28 °C with continuous shaking in a Bioscreen C system (MBR). The OD600 was measured every 30 min to determine the turbidity of the cultures. The assays were carried out at least twice.

A Live/Dead staining method based on two nucleic acid probes was followed to differentiate and quantify live and dead cells in bacterial cultures, as previously described [38]. The dyes used were propidium iodide PI, red-colored, which only enters membrane-compromised cells, and SYTO13 (Invitrogen), green-coloured, which is membrane-permeant and stains both viable and non-viable cells. Exponential and stationary phase cells were harvested by centrifugation, washed and adjusted to an OD600 of 0.1. Then, the cell suspensions were incubated with 10 mM NaCl for live cells or 70% isopropyl alcohol for dead cells. A standard curve with various proportions of live/dead cells was prepared, and both, the standards and the samples were stained according to the manufacturer indications. The staining solution (0.01 mM SYTO13 and 0.06 mM PI) was mixed with the bacterial suspensions in a 1:1 ratio in a 96-well microplate (Greiner). The samples were incubated in the dark for 15 min before measuring fluorescence in a multiplate reader VarioskanLUX (ThermoScientific, Waltham, MA, USA). Intensities of green (510 nm) and red (630 nm) emission were recorded after excitation at 485 nm and 530 nm, respectively. The green to red fluorescence emission ratio, which is proportional to the relative number of live bacteria, was calculated.

### 2.8. Bioinformatics Analyses

The Universal Protein database UniProt was used for protein biological and molecular functions, to retrieve sequences and IDs, and other searches of protein information (http://www.uniprot.org; accessed on 22 November 2022). Prediction of subcellular localization was done using PSORTb version 3.0.3 tools (https://www.psort.org; accessed on 22 November 2022) and the moonlighting databases used were MoonProt (http://www.moonlightingproteins.org; accessed on 22 November 2022) and MultitaskProtDB (http://wallace.uab.es/multitask/; accessed on 22 November 2022).

## 3. Results

### 3.1. Cyclic Diguanylate-Dependent Extracellular Proteome of Rhizobium etli

We studied the relative abundance of proteins in the culture supernatants of a *R. etli* strain expressing high contents of cdG (strain LR101), compared to an isogenic derivative expressing physiological cdG levels (strain LR102). This study was performed following two different proteomic methodologies: classical 2D-GE proteomics and i-TRAQ labelling coupled to quantitative MS-based proteomics.

In total, 128 distinct proteins were identified from more than 200 2D-GE spots analysed (Table S1). Frequently the same protein could be identified in different spots, sometimes with opposite trends. For instance, the hypothetical protein RHE_CH00301 was identified in 10 separate spots, 6 with increased abundance (IA) and 4 with reduced abundance (RA) in the LR101 supernatants. Glyceraldehyde 3-phosphate dehydrogenase was identified in four different spots, three of which were IA in the supernatants of the LR101 (# eD69, # eD179 and # eD180) whereas the fourth (# e107) was RA (Table S1). Flagellins were identified in 17 spots, of which 9 corresponded to FlaCch1, 5 to FlaCch2 and 3 spots to FlaCch3 (Table S1).

A total of 64 spots corresponded to proteins related with transport systems, mainly substrate-binding proteins of ABC transporters, most of which appear as RA in the LR101 supernatants. For instance, the solute-binding protein RHE_CH04006 was identified in four spots, three of which were RA (e108, e110, eD252) and another one IA in the LR101 culture supernatants (eD110; Table S1). The finding of one same protein in separate spots was indicative of proteoforms with different molecular weight and/or isoelectric points (pI). However, differences in the relative abundance of proteoforms hindered the quantitative analysis of the cdG dependent extracellular proteome.

**Figure 1:**
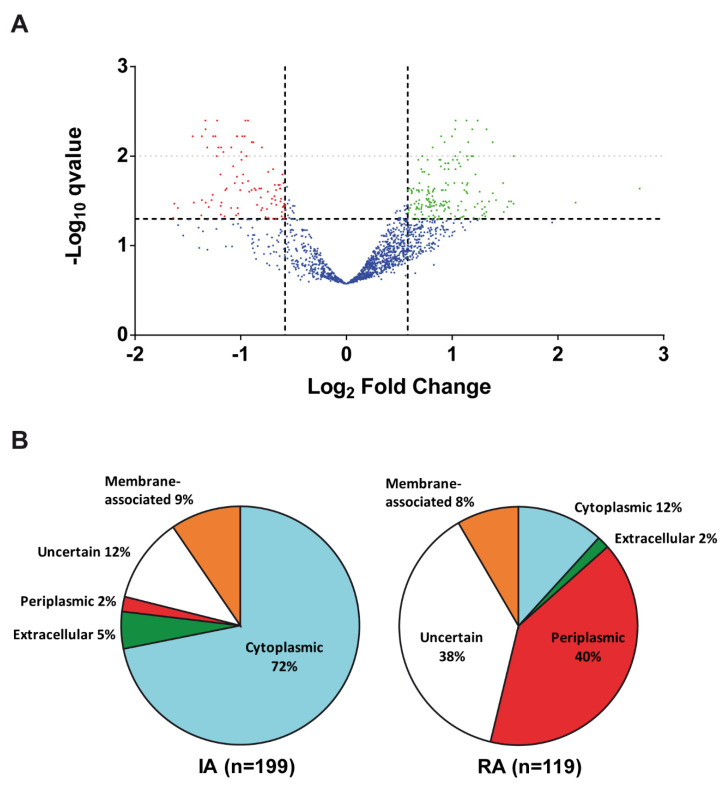
Extracellular proteome of *R. etli* CFN42 under high intracellular levels of c-di-GMP. (**A**) The increased abundance (IA, green dots) and reduced abundance (RA; red dots) culture supernatants proteins between LR101 (high levels of cdG) and LR102 (physiological levels of cdG) strains, identified by iTRAQ labelling and coupled with MS-based proteomics are represented. Identified proteins qualified as statistically Confident (q-value < 0.01 and ±1.5 fold-change) and Likely (q-value < 0.05 and ±1.5 fold-change) differentially abundant are represented as faint and strong dotted lines, respectively. (**B**) Predicted subcellular localization of the differentially abundant (IA and RA) proteins identified under high cdG (LR101) by 2D-GE proteomics and iTRAQ labelling coupled with quantitative MS-based proteomics.

Quantitative iTRAQ labelling coupled with MS-based proteomics allowed for the identification of thousands of peptides and hundreds of proteins (Table S2), as represented in a volcano plot (Figure 1A). However, only those qualified as statistically Confident or Likely differential abundance were considered (Table S2). Exceptions were several proteins qualified as “putative” (either up or down), which had also been identified in the previous 2D-GE analysis. Despite the methodological dissimilarities, there were striking coincidences between both proteomics procedures, thus providing further confidence in the results. For instance, both proteomic approaches identified the same proteins including many transport proteins, flagellins, elongation factor Tu, transcription elongation factor GreA, chaperonin GroEL or enzymes such as malate dehydrogenase, glyceraldehyde 3-phosphate dehydrogenase and cysteine synthase (Tables S1 and S2).

Considering the results from both proteomic approaches (Tables S1 and S2), 318 proteins were considered as differentially abundant (DA) in the culture supernatants of the high cdG strain compared with the control strain. Of these, 199 proteins (62.5%) presented increased abundance (IA) and 119 (37.5%) displayed reduced abundance (RA) in the supernatants of the high cdG strain (Figure 1B).

We have also studied the predicted subcellular location and likely function (based on analysis of protein domains, sequence homologies and literature reports) of each individual DA protein. Regarding subcellular localization, only 5% of IA proteins in culture supernatants of the high cdG strain were likely extracellular proteins, whereas nearly 72% were predicted to be cytoplasmatic, 9% were predicted to be membrane-associated and 2% periplasmic; the remaining 12% were proteins of uncertain location (Figure 1B). On the other hand, RA proteins in the culture supernatants of the high cdG strain were very different from the IA proteins, and nearly 40% were predicted to be periplasmic, 12% cytoplasmic, 8% membrane-associated and 2% extracellular. A significant 38% were of uncertain subcellular localization.

Gene Ontology (GO) functional analysis of DA proteins showed that a majority of RA proteins were of unknown function or involved in transport processes (Figure S1). On the contrary, most IA proteins appeared involved in housekeeping processes such as protein synthesis, carbon or nitrogen metabolisms (Figure S1). A more detailed functional study was carried out with every one of the proteins which had a known or predicted function.

Some of the DA proteins in the supernatants of the cdG strain have a predicted extracellular location, and some could even participate in processes related to or known to be regulated by cdG, such as adhesion/aggregation and biofilm formation. Among these was an ortholog of a rhizobial adhesion protein (RapAch, RHE_CH03231), which in *R. leguminosarum* is known to be secreted by the Type-1 Secretion System (T1SS) PrsDE [39,40]. There were also two large extracellular proteins (RHE_CH02633; RHE_CH02634) containing multiple putative cadherin-like domains (PF17803; IPR 040853), VCBS repeats (TIGR01965) and RTX Ca-binding domains (PF00353, IPR001343). Given the domain predictions, these two proteins likely represent novel adhesins (Table 1). There are no previous reports that cdG promotes the export of any of these adhesins in rhizobia. Contrary to RapAch, two other Rap proteins, RapB1 and RapB2, displayed RA in the cdG supernatants (Table 1), which suggests that cdG differentially affects the production of distinct rhizobial adhesion proteins. We also found IA several flagellins (FlaCch2, FlaCch3, FlaCch4, FlaCch5, FlaCe) as well as the flagellar hook-related proteins FlgE, FlgK and FlgL, and the distal rod proteins FlgG and FlgD (Table 1). We have previously reported that high cdG completely blocks the motility of *R. etli* [41]; thus, our results suggest that cdG may not equally repress flagellin secretion. In *Sinorhizobium meliloti*, elevated cdG contents also inhibit motility without affecting flagellar gene expression [24,42]. In this bacterium, cdG negative regulation of motility involves cdG binding to the PilZ-domain protein McrA, whereas flagella and pili have been found to contribute to cdG-dependent biofilm formation [43].

Other proteins in this group do not have a predicted extracellular location but could participate in extracellular processes. For instance, RkpK is likely required for LPS and KPS biosynthesis [45] and PssP for polymerization of an acidic exopolysaccharide [46], whereas TrbB and TrbE are components of the T4SS required for conjugal transfer of plasmid pCFN42a ([47]; Table 1). Contrary to the above, the extracellular proteins PssO (involved in exopolysaccharide export) and PlyA1 (an extracellular glycanase; [48]) were both RA. The peptidoglycan-binding YkuD-like protein RHE_CH01507 and two putative murein transglycosidases (RHE_CH02869 and RHE_CH04101) also displayed RA in the supernatants of the cdG strain LR101 (Table 1).

The largest functional group (63) of DA proteins in the supernatants of the cdG strain were related to the transport across membranes, especially ABC-type transporters. A great majority (53) of these transport proteins displayed RA and mainly corresponded to periplasmic substrate-binding components of ABC transporters. In contrast, only 10 transport-related proteins displayed IA, and these were mostly inner membrane-associated proteins (Table 1). There were several examples of components of a given transporter that showed opposite behaviours. For instance, the ATP-binding component of a phosphate transporter PstB was IA, whereas the periplasmic component PstS appeared as RA; the zinc transporter ZnuC was IA but its periplasmic partner ZnuA was RA; and the Tol translocation component TolQ was IA whereas TolB displayed RA (Table 1).

As mentioned above, many of the IA proteins in the supernatants of the cdG strain are predicted to be cytosolic proteins. The most abundant functional group of cytoplasmic proteins were involved in protein synthesis or modification (Table 1). These included 14 large (50S) and 9 small (30S) ribosomal subunits, various ribosome-associated proteins such as elongation factors EF-Tu (Tuf) and EF-G (FusA2) and the ribosome binding ATPase YchF, as well as several proteases and protein chaperones.

Ribosomal proteins are frequently found as part of the surface or extracellular proteomes of bacteria [44,49,50]. In cyanobacteria, secretion of ribosomal and other cytoplasmic proteins has been shown to require the type IV pili assembly machinery, the Hfq RNA chaperone and a EbsA protein involved in biofilm suppression [50].

Elongation factor thermo unstable EF-Tu is one of the most abundant and universally conserved proteins in bacteria and eukaryotes. It is a GTPase that ensures translational accuracy by catalysing the binding of aminoacyl-tRNA to the aminoacyl site (A-site) of the ribosome. In addition to its primary role, EF-Tu has been reported to play various other roles in diverse organisms [51]. EF-Tu is frequently found outside the cell of bacterial pathogens, where it can play a role in virulence [51,52]. EF-Tu is also a well-described Pathogen Associated Molecular Pattern (PAMP) molecule for plants. An EF-Tu receptor (EFR) found within *Brassica* lineages recognises highly conserved N-terminal 18 amino acids (elf18) in the native EF-Tu molecule [53]. Furthermore, EF-G has been proposed as a cytoplasmic/surface moonlighting protein in bacteria [44].

Several subunits of cytoplasmic proteases were also found to be IA, including the ClpP2, ClpP3, ClpA and ClpX components of CLP, the Lon and the HslUV (ClpQY) proteases and a putative prolyl oligopeptidase (PtrB). The membrane-anchored metalloprotease FtsH and its regulators HflKC, as well as the membrane-associated insertase YidC, were also found to be IA in the supernatants of the cdG strain (Table 1). Contrary to the increased abundance of those proteases, the periplasmic putative protease inhibitor RHE_CH01654 displayed RA. Components of some of these proteases have been found associated to the cell surface of several bacteria and proposed as intracellular/surface moonlighting proteins [44]. Molecular chaperones such as GroEL (GroL1, GroL2 and GroL4), DnaK and Tig (trigger factor) were also found IA in the supernatants of the cdG strain. Contrary to all the above proteins, a putative methionyl-tRNA formyltransferase protein Fmt was found RA (Table 1). DnaK and GroEL chaperones have also been reported to moonlight in several bacteria, with a second role in adhesion (see http://www.moonlightingproteins.org/proteins/ accessed on 22 November 2022). GroEL has additionally been described as an insect toxin in endosymbiotic *Enterobacter aerogenes* [54]. Trigger factor has also been proposed as an intracellular/surface moonlighting protein in bacteria [44].

A large group of supernatant IA proteins participate in Carbon metabolism, particularly enzymes of the glycolysis (Enolase, Fructokinase, Phosphofructokinase, Fructose-bisphosphate aldolase, Glyceraldehyde 3-phosphate dehydrogenase, Glucokinase); the tricarboxylic acid cycle (TCA; SucA and SucB components of α-ketoglutarate dehydrogenase, SucC and SucD of Succinyl-CoA synthetase; Fumarate hydratase and Malate dehydrogenase, Citrate synthase); and the pyruvate dehydrogenase Pdh complex (Table 1). Most these glycolytic and TCA proteins have been previously reported as moonlighting or putative moonlighting proteins (Wang and Jeffery, 2016; see http://www.moonlightingproteins.org/proteins/ accessed on 22 November 2022). Other proteins in this group were phosphoenolpyruvate carboxykinase (PckA), a putative phospho-2-dehydro-3-deoxygluconate aldolase (Eda) and a Gluconokinase (KdgK), involved in the pentose-phosphate pathway, a glucose-1-phosphate adenylyltransferase (GlgC), likely involved in glycogen biosynthesis, a putative xylose isomerase (XylA) and an aldehyde dehydrogenase RHE_CH03723. On the contrary, a putative sugar isomerase/dehydratase (GalE2) and two cytoplasmic carbonic anhydrases (CynT; RHE_PD00192) displayed RA in the extracellular proteomes of the cdG strain (Table 1).

Another important group of IA proteins were related to Nitrogen metabolism (Table 1). The ammonium transporter AmtB, the type-1 glutamine synthetases (GS) GlnA1 and GlnA2 and 17 proteins related to biosynthesis of several amino acids: serine (SerA, SerC), threonine (ThrA, AatCch), methionine and cysteine (MetK, MetZ, MdeAf, CysK1, AhcY), isoleucine/valine (IlvV, IlvDch2, IlvE2), arginine (ArgF), aspartate (AatAch), histidine (HisB) and tyrosine (TyrC). Proteins involved in both biosynthesis (AldA) and catabolism (DadA) of alanine were also found IA extracellularly. Several proteins related to nitrogen-fixing symbiosis with legumes also showed as IA in the extracellular proteome: the regulators NifA, NtrY and ChvI, and the nodulation proteins NodT and NoeJ. On the contrary, the NodD-like transcriptional regulator RHE_PD00316 was found RA (Table 1).

A significant group of proteins were related with nucleotide, RNA and DNA metabolisms (Table 1). Adenylosuccinate synthetase (PurA), Adenylosuccinate lyase (PurB) and Inosine 5′-monophosphate dehydrogenase (GuaB), involved in purine metabolism, were found IA, as well as Uracil phosphoribosyltransferase (Upp), Uridylate kinase (PyrH) and a putative Cytosine deaminase (CodAb) participating in pyrimidine metabolism. Contrarily, the cyclic nucleotide phosphodiesterase CpdB and Adenylate cyclase CyaJ were found RA.

Several proteins related to transcription and RNA processing were found IA in the supernatants of the cdG strain (Table 1): the RNA polymerase structural subunits (RpoA, RpoB, RpoC) and the RpoD σ70 factor, the transcription factor NusA, the transcription-repair coupling factor (TRCF, Mfd) and the mRNA degradation polyribonucleotide nucleotidyltransferase (Pnp). On the contrary, the transcription elongation factor GreA and the Nudix hydrolase-like IalA displayed RA. Regarding DNA processing, DNA polymerase III beta chain (DnaN), helicase MgpS, DNA gyrase subunits GyrA and GyrB, DNA repair recombinase RecA and the DNA topoisomerase subunit ParC were found IA, whereas the histone-like protein Hrm appeared as RA (Table 1). Many of these proteins have also been proposed as candidate moonlighting proteins in other bacteria [44].

Several proteins involved in electron transport and energy production were found to be IA extracellularly: the ATP synthase α, β, γ, δ and ε subunits; components of the cytochrome bc1 membrane complex (FbcC, FbcF) and cytochrome c oxidase (CtaC); the electron transfer flavoprotein EtfAf, a putative type-2 NADH dehydrogenase (NdhCh) and the putative H+-pump pyrophosphatase RrpP. On the contrary, the cytochrome c-like proteins CycF and RHE_CH02618 displayed RA (Table 1).

Several proteins involved in lipid metabolism also appeared IA extracellularly. These included the PhbA and PhbB proteins involved in the synthesis of the carbon storage polymer poly-β-hydroxybutyrate (PHB), the fatty acid biosynthesis proteins FabA, FabF1, FabI1, RHE_CH01048, RHE_CH01307 and RHE_CH03352, and the fatty acid degradation proteins acyl-CoA dehydrogenase Acd1 and 3-hydroxybutyryl-CoA dehydrogenase HbdA.

Regarding metabolism of cofactors and metals, a thiamine biosynthesis protein ThiO, cobalamine biosynthesis CobN and CobS, the Fe-S cluster carrier protein RHE_CH00859, VbsA and VbsL required for biosynthesis of the siderophore vicibactin [55] and the bacterioferritin-like protein Bfr were all found as IA (Table 1).

Related to stress responses, the osmotically inducible peroxidase OsmC [56] was IA, whereas the oxidative stress related protein NerA [57] was RA.

### 3.2. High cdG Levels Do Not Enhance Cell Lysis nor MV Formation

Growth curves of both strains, LR101 and LR102, displayed no significant differences either in rich medium TY or minimal medium MMY (Figure S2). For both strains, the MM cultures reached significantly lower densities than in TY. Despite the inability to produce cellulose, both strains still showed slight aggregation/flocculation in MMY, specially the cdG strain LR101, which may have interfered with turbidity reads. In addition, fluorescent live/dead viability assays were carried out as described in Materials and Methods. Cultures in the same media and conditions as the proteomic studies were used to evaluate the live/dead cell ratios. For both strains LR101 and LR102, cells in the exponential growth phase (DO_600_ = 0.1) were used as control live cells, whereas cells exposed to isopropyl alcohol were used as control dead cells. In both strains, the live/dead ratio was above 90% along the growth curve, with no significant differences between them. In both cases, the live/dead cell ratio only started to decrease in the late stationary phase.

In order to determine if cdG could have an effect in the production of membrane vesicles (MVs), isolation and quantification of MVs in both bacterial strains was carried out. Direct visualization and quantification of MVs was hindered by the high amounts of viscous material, probably polysaccharides present in the supernatants of cell cultures, especially from the cdG strain LR101. MV production was then indirectly measured by quantification of total lipid contents with the fluorescent probe FM4-64. Relative fluorescence units (RFU) of supernatants of the strains LR101 (2.08 × 10^3^ ± 3.18 × 10^2^) and LR102 (2.30 × 10^3^ ± 1.06 × 10^2^) were not statistically nor significantly different (*p* > 0.05; Tukey HSD test). This indicated that cdG does not promote enhanced production of MVs in this bacterium.

### 3.3. cdG Promotes Exportation of the Cytoplasmic Proteins EF-Tu and Gap

In order to verify cdG-enhanced exportation in the cdG strain LR101, we performed Western blot (WB) assays of two important cytoplasmic proteins, EF-Tu and Gap. These two cytoplasmic proteins were chosen for several reasons. EF-Tu displayed IA in both 2D-GE and iTRAQ proteomics of culture supernatants. Gap was particularly interesting because iTRAQ proteomics draw it as putative up (Table S2); however, it was identified in various differential 2D-GE spots in the LR101 supernatants (Table S1). Both proteins have been previously detected in the culture supernatants of wild-type *R. etli* under various growth conditions [58,59]. In addition, both EF-Tu and Gap proteins have been repeatedly found extracellularly in several bacteria, and both have been catalogued as moonlighting proteins [51,60].

We carried out immunoblots of proteins extracts of the cell pellets and the supernatants of cultures of both LR101 and LR102 strains. Culture conditions were the same as those described for the proteomic studies. EF-Tu was revealed with a commercial antibody raised against a peptide of *E. coli* EF-Tu, which is highly conserved amongst Gram-negative EF-Tu proteins. A policlonal antibody raised against a synthetic peptide derived from the *R. etli* Gap protein sequence was used for Gap immunodetection (see M and M).

**Figure 2:**
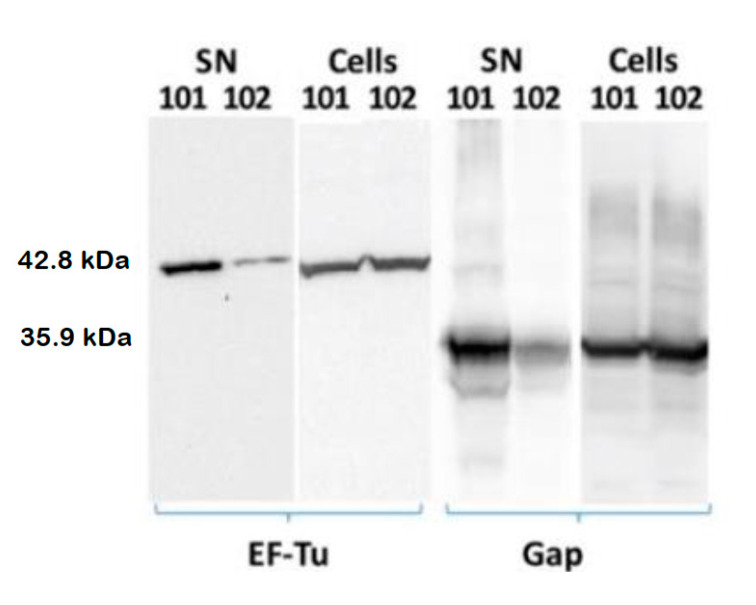
Immunodetection of proteins EF-Tu and Gap in supernatants (SN) and cell extracts of cultures of *R. etli* strains LR101 and LR102. In total, 20 µg of total protein was loaded into each lane.

As expected, both proteins could be immunodetected in the cell fractions and in the supernatants of both LR101 and LR102 cultures, albeit protein abundances were much higher in the cell extracts (Figure 2). However, we could observe a significantly increased abundance of both proteins in the supernatants of the cdG strain LR101, compared to the isogenic LR102 (Figure 2). In contrast, no significant differences were detected for the cell fractions of the two strains, thus supporting the results of proteomic experiments. The data indicate that high cdG levels do not affect the cellular contents of these two cytoplasmic proteins but promote their exportation to the cell exterior.

### 3.4. Subcellular Localization of R. etli Gap Protein

**Figure 3:**
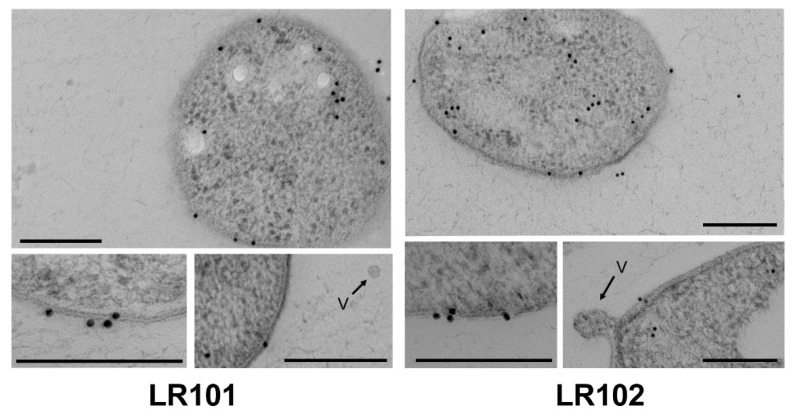
Subcellular localization of Gap in *R. etli* by immunocytochemistry and electron microscopy. In both strains, LR101 and LR102, colloidal gold particles, were localized in the cytoplasm of bacterial cells, and are associated with the inner and outer membrane in the periplasmic space and outside the cells. No gold particles were localized in vesicles (V). All scale bars = 200 nm.

Immunocytochemical experiments were performed to verify the subcellular localization of the *R. etli* Gap protein. Ultrathin cryosections were obtained and incubated with the anti-Gap specific antibody. In both strains, LR101 and LR102, the immunolabelling revealed the presence of Gap mainly in the cytoplasm, but also associated to the inner and outer membranes and the periplasm (Figure 3). The finding of Gap in the cytoplasm is consistent with its intracellular function in sugar metabolism. However, detection of the protein clearly associated with the inner and outer membranes and even outside the cells suggests that specific Gap exportation occurs in both strains. Unfortunately, this technique does not allow for establishing quantitative differences between the strains.

### 3.5. Extracellular Gap Proteoforms Promoted by c-di-GMP

**Figure 4:**
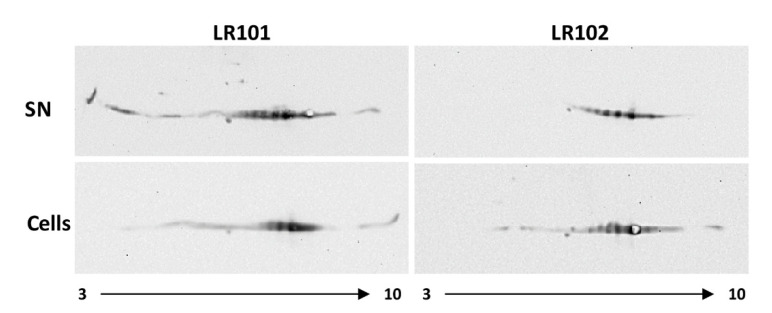
Two-dimensional-GE Western Blot and Immunodetection of *R. etli* Gap. Immunodetection of Gap protein was performed with polyclonal antibodies against *R. etli* Gap. 80 µg of total proteins from supernatants (SN) or cell extracts from cultures of *R. etli* LR101 and LR102 were loaded, except for LR102-SN, for which 100 µg of protein was loaded. The pH gradient (3–10) applied in the first dimension is indicated.

We carried out bidimensional separation of total proteins in both cell-associated and supernatant fractions from LR101 and LR102 cultures, followed by Western blot and specific immunodetection of the Gap protein. In all samples, multiple Gap proteoforms could be separated and detected (Figure 4), which is suggestive of posttranslational modifications (PTMs) of the protein. This would not be surprising since Gap has been reported to bear a variety of PTMs in various organisms [61,62].

Intriguingly, the abundance and especially the number of Gap proteoforms appeared higher in the culture supernatants than in the cell fractions. This was particularly true for the cdG strain LR101 supernatants, where exclusive proteoforms displaying much lower pI than that predicted from the Gap protein sequence (6,87) were detected (Figure 4). The absence of these proteoforms from the cell fractions suggest that specific Gap proteoforms could be tagged for exportation. The results indicated that cdG promotes exportation of the Gap protein to the cell exterior, and that this exportation could involve particular proteoforms.

## 4. Discussion

In this work, we have explored changes provoked by high cdG levels in the extracellular proteome of the nitrogen-fixing symbiont *R. etli* CFN42. There were several surprising, unexpected results, but perhaps the most striking result was that much of the cdG- dependent extracellular proteome was composed mainly by proteins that are otherwise known or predicted to have a cytoplasmic localization. Several data support that the changes in the amounts of cytoplasmic proteins in the extracellular proteome are not a consequence of stochastic or fortuitous events, but likely represent a physiological response to the high levels of intracellular cdG in the strain LR101.

High cdG levels do not seem to significantly affect cell growth rates, neither in rich nor in minimal media. We have also verified that the high cdG strain LR101 displays similar death rates than the control LR102, which discards stochastic phenomena such as differences in cell death or lysis.

The *R. etli* CFN42 genome contains nearly 6000 protein-coding genes. However, only about 27% of total proteins (1646) were identified in the culture supernatants, of which less than 15% (318) displayed significant differential abundance in the supernatants of the high cdG strain LR101. Although 62.5% of these DA proteins were IA, a significant 37.5% displayed RA in the LR101 supernatants.

Out of the 318 proteins that were considered DA in the LR101 supernatants, some 157 were proteins known or predicted to have a cytoplasmic localization. Most these cytoplasmic proteins displayed IA in the supernatants of the LR101 cultures; however, at least 14 displayed RA. The majority of these cytoplasmic proteins have a known or likely housekeeping function, many involved in protein synthesis and modification, carbon, nitrogen and energy metabolisms. A large fraction (52%, 83 proteins) of these cytoplasmic proteins whose export appears promoted by cdG are proteins that have been reported as moonlighting or candidate moonlighting proteins in other organisms [44].

Moonlighting proteins comprise a class of multifunctional proteins that perform multiple but often unrelated functions. Hundreds of moonlighting proteins have been reported in all classes of organisms, prokaryotes, eukaryotes and viruses (http://www.moonlightingproteins.org/ accessed on 22 November 2022; http://wallace.uab.es/multitask/ accessed on 22 November 2022). Frequently, moonlighting involves changes of protein subcellular location. Thus, many cytoplasmic metabolic proteins and chaperones have been shown to have other roles, unrelated to their well-known enzymatic activities, when localized outside the cell [63,64,65,66]. These are called intracellular/surface moonlighting proteins and play important roles in bacteria-host interactions, since many of them have been shown to bind to specific host proteins [44,67]. Several authors support that cytoplasmic proteins can be secreted outside the cell by yet unknown, non-classical secretion systems [20,21,22]. Non-classical protein secretion has been used as a biotechnological tool to overproduce extracellular recombinant proteins [19].

We have confirmed that cdG-promoted exportation for two of these housekeeping cytoplasmic proteins: translation elongation factor EF-Tu and Glyceraldehyde-3-P dehydrogenase (Gap), both after WB, immunodetection and quantification. These two proteins displayed IA in the culture supernatants of the cdG strain, but no changes in the cell fraction. Both EF-Tu and Gap are frequently described as extracellular proteins in many bacteria under normal growth conditions, including *R. etli* [58,59]. EF-Tu and Gap are also two of the best-known bacterial moonlighting or multifunctional proteins, with a frequent role in bacterial adhesion when located outside the cell [44,52,61], in addition to their important housekeeping cytoplasmic functions. What is totally novel is that their exportation is promoted by the second messenger c-di-GMP.

Electron microscopy (EM) has allowed us to localize the *R. etli* Gap protein in different subcellular localizations: inside the cytoplasm, outside the cell and also associated with the inner and outer membranes, suggesting an active process of exportation that is present in the wild-type strain but would be increased by high levels of cdG. Evidence suggests that the cdG-promoted export of this protein is not via MVs. High cdG levels do not seem to promote enhanced production of MVs, however, we cannot discard that cdG may provoke changes in MV protein cargos. We have found evidence that cdG promotes a substantial increase in the number of Gap proteoforms that can be detected after 2D-GE. Particularly striking was the detection of certain proteoforms exclusively in the culture supernatants, but not in the cell extracts of the cdG strain. This suggests the specific dedication of such proteoforms for exportation outside the cell. The existence of multiple proteoforms for the same protein can be indicative of post-translational modifications (PTMs). PTMs are chemical modifications that can increase the complexity of the proteome by regulating the activity, localization or interactions of proteins with other molecules [68,69]. PTMs can be reversible or irreversible and may occur at any time during the life of a protein. Common protein PTMs involve phosphorylation, glycosylation, acylation or methylation; however, nearly 300 different types of PTM have been described [70,71]. Glyceraldehyde 3-phosphate dehydrogenase from various sources has been reported to be subject to various types of PTMs [61,62]. In the case of moonlighting proteins, there are examples of PTMs causing a switch between two of the functions of the protein, thus serving as a toggle between functions [72].

The most widely known protein PTM is phosphorylation. In a recent report, Yagüe et al. [73] made a compilation of protein phosphorylations in bacteria. They compared 38 bacterial phosphoproteomes and identified 29 orthologues present in at least four phosphoproteomes. Among these, 29 phosphoproteins are Gap, Eno, several 50S and 30S ribosomal proteins, EF-Tu, RpoC, the ATP synthase α subunit, the chaperones GroEL and DnaK [73], all cytoplasmic proteins that we have identified in the supernatants of the *R. etli* strain expressing elevated cdG levels.

There are few precedents of a relationship between cdG regulation and protein PTMs. Xu et al. [74] reported that cdG binds the deacetylase CodB to modulate its activity. CodB activity impacts diverse processes in *E. coli*, such as the synthesis of acetyl-CoA (a central intermediate in energy and lipid metabolism), or the chemotaxis regulator CheY [74]. On another hand, the activity of the protein RimK in *Pseudomonas* is modulated by the trigger enzyme RimA and the protease RimB, whose activities seem coordinated by cdG. RimK is an ATP-dependent glutamyl ligase that adds glutamate residues to the C-terminus of ribosomal protein RpsF, thereby inducing specific effects on both ribosome protein complement and function. At the phenotypic level, this RpsF modification determines important aspects of rhizosphere colonisation through proteome remodelling [75,76].

## 5. Conclusions

Our results show that elevated cdG intracellular levels determine significant changes in the extracellular proteome of *R. etli*. As expected, cdG promoted the exportation of extracellular proteins (adhesins, flagellins, etc.) that likely participate in cdG-regulated processes such as adhesion and biofilm formation. However, unexpectedly, cdG also promoted the selective exportation of otherwise known or predicted cytoplasmic proteins. Nearly 50% of these cytoplasmic proteins have been previously described as moonlighting or candidate moonlighting proteins in other organisms, often found extracellularly or at the cell surface of bacteria. Our data support that the cdG-dependent exportation of the *R. etli* Gap protein involves an active mechanism and dedicated Gap proteoforms that likely represent yet unknown post-translational modifications of the protein. Thus, our results reveal a link of cdG regulation with the post-translational modification and export of cytoplasmic proteins, and possibly with the phenomenon of protein moonlighting. Our next goals are the characterization of cdG-promoted extracellular Gap proteoforms and associated PTMs, as well as determining the moonlighting functions of the *R. etli* Gap protein.

## Figures and Tables

**Table 1:** Functional classification of Differential Abundance (DA) proteins in the culture supernatants of the cdG strain LR101.

Category	Function	Differential Abundance Proteins	
		Increased Abundance (IA)	Reduced Abundance (RA)
**Extracellular Function and Structural**	Aggregation/Adhesion	RapAch, RHE_CH02633, RHE_CH02634	RapB1, RapB2
	Flagella/Motility	FlaCch2, FlaCch3, FlaCch4, FlaCch5, FlaCe, FlgKch, FlgEch, FlgD, FlgG, FlgL	
	Peptidoglycan		RHE_CH02869, RHE_CH04101, RHE_CH01507
	Polysaccharide	RkpK, PssP	PlyA1, PssO
	Conjugation	TrbB, TrbE	
**Protein Synthesis and Modifications**	Chaperones	**GroL1**, **GroL2**, **GroL4**, Tig, YidC, **DnaK**,	PpiD2
	Proteases	ClpA, ClpP2, ClpP3, ClpX, **FtsH**, HflC, HflK, HslU, HslV, Lon, PtrB	RHE_CH01654
	Translation	GatA, GatB, **Tuf**, **FusA**, **GltX**, **RpsA**, RpsB, **RpsC**, **RpsD**, RpsE, **RpsH**, RpsK, RpsQ, RpsU1, **RplA**, **RplB**, RplC, **RplD**, **RplE**, RplI, **RplN**, RplP, RplS, RplU, RplV, RplX, RplY, RpmA, YchF	Fmt
**Carbon Metabolism**	Glycolysis/TCA	**Eno**, Frk, Pfk, **FbaB**, **Gap**, Glk, PdhA1, **PdhA2**, **PdhB**, **LpdAch1 SucA**, SucB, SucC, SucD, **Mdh**, **GltA**, **FumC**	
	Others	PckA, GlgC, Eda, KdgK, XylA, RHE_CH03723	GalE2, CynT, RHE_PD00192
**Nucleid Acid Metabolism**	Transcription	NusA, RpoA, **RpoB**, RpoC, RpoD, Mfd, Pnp	GreA, IalA,
	Purin and Pyrimidine Metabolism	PurA, PurB, GuaB, **Upp**, **PyrH**, CodAb	**CpdB**, CyaJ
	Replication/Recombination	DnaN, **MgpS**, GyrA, GyrB, ParC, RecA	
	Others		Hrm
**Nitrogen Metabolism**	Amino Acid Metabolism	AldA, DadA, **ArgF**, SerA, SerC, ThrA, TyrC, HisB, **IlvC**, IlvDch2, IlvE2, MetK, MetZ, **CysK1**, MdeAf, AhcY, AatAch, AatCch,	RHE_PF00434
	BNF-Symbiosis	NifA, NodTch, NtrY, NoeJ, ChvI	NodD-like RHE_PD00316
	Nitrogen Assimilation	GlnA1, GlnA2, AmtB	
**Lipid Metabolism**	Lipid biosynthesis and degradation	HbdA, PhbAch, PhbB, FabA, FabF1, FabI1, Acd1, RHE_CH01048, RHE_CH03352, RHE_CH01307	
**Energy Production/Electron transfer**	ATP synthesis and electron transfer	AtpA, AtpC, AtpD, AtpF2, AtpG, AtpH, FbcC, FbcF, RrpP, EtfAf, CtaC, NdhCh	CycF, RHE_CH02618
**Cofactors–Metals**	Biosynthesis of cofactors and metal metabolism	Bfr, CobN, CobS, ThiO, RHE_CH00859	
	Biosynthesis of siderophores	VbsA, VbsL	
**Stress**	Stress responses	OsmC	NerA
**Transport**	Transporters	AglK, SecD1, PstB, ZnuC, MntH, TolQ, SufC, RHE_CH00008, RHE_CH02346, RHE_PB00094	PstS, TolB, UgpBc, UgpBch1, XylF, DppAch1, DppAch2, DppAch3, RbsBch3, AapJ, BraC1, BraC2, OppA, AfuA1, AfuA3, HmuT, OccT, ModA, PotF, MexE1, ZnuA, RHE_CH00971, RHE_PC00008, RHE_PC00118, RHE_PC00160, RHE_CH02418, RHE_PF00186, RHE_CH01465, RHE_CH04006, RHE_PF00410, RHE_CH02293, RHE_CH03027, RHE_CH03963, RHE_CH03445, RHE_PB00126, RHE_CH00175, RHE_PF00269, RHE_CH02683, RHE_CH02890, RHE_PE00259, RHE_PF00068, RHE_CH00492, RHE_CH01210, RHE_CH02898, RHE_PF00091, RHE_PB00025, RHE_PF00321, RHE_PB00139, RHE_PF00395, RHE_CH00485, RHE_PC00167, RHE_CH03866, RHE_CH02084

Bold letters indicate proteins reported as moonlighting in other organisms (see http://www.moonlightingproteins.org/proteins/ accessed on 22 November 2022). Underlined are proteins proposed as candidate Intracellular/Surface moonlighting proteins in other bacteria [44].

## Data Availability

Not applicable.

## Supplementary Materials

The following supporting information can be downloaded at: https://www.mdpi.com/article/10.3390/biology12010044/s1, Figure S1: Gene Ontology (GO) classification of proteins; Figure S2: Growth curves of the *R. etli* strains LR101 and LR102. Table S1: ExtracellularSpots-2D-GE; Table S2: Extracellular-iTRAQ.
