# Supplementary material for: Impact of c-di-GMP on the Extracellular Proteome of Rhizobium etli
Source: Biology (Basel). 2022 Dec 26;12(1):44. doi: 10.3390/biology12010044 (PMC9855851; doi:10.3390/biology12010044)
Supplement: Supplementary file 1 [file biology-12-00044-s001.zip › Figures S1 and S2.pdf]

biology

MDPI

Supplementary Materials

Impact of c-di-GMP on the Extracellular Proteome of *Rhizobium etli*

María J. Lorite [1], Ariana Casas-Román [1], Lourdes Girard [2], Sergio Encarnación [2], Natalia Díaz-Garrido [3,†], Josefa Badía [3,4], Laura Baldomá [3], Daniel Pérez-Mendoza [1] and Juan Sanjuán [1,*]

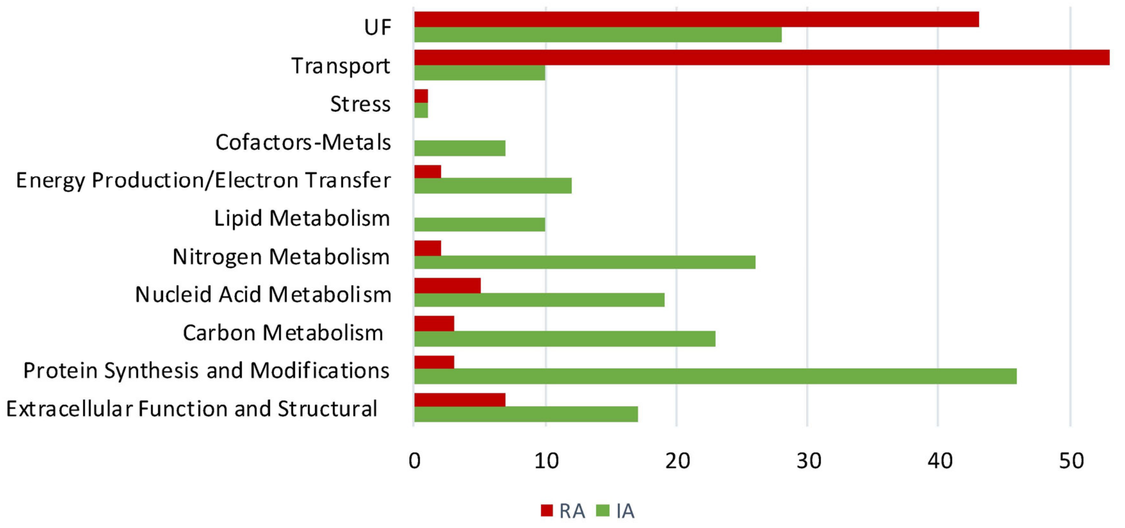

Figure S1. Gene Ontology (GO) classification of proteins displaying differential abundance in the supernatants of the high cdG strain LR101. IA increased abundance, RA reduced abundance.

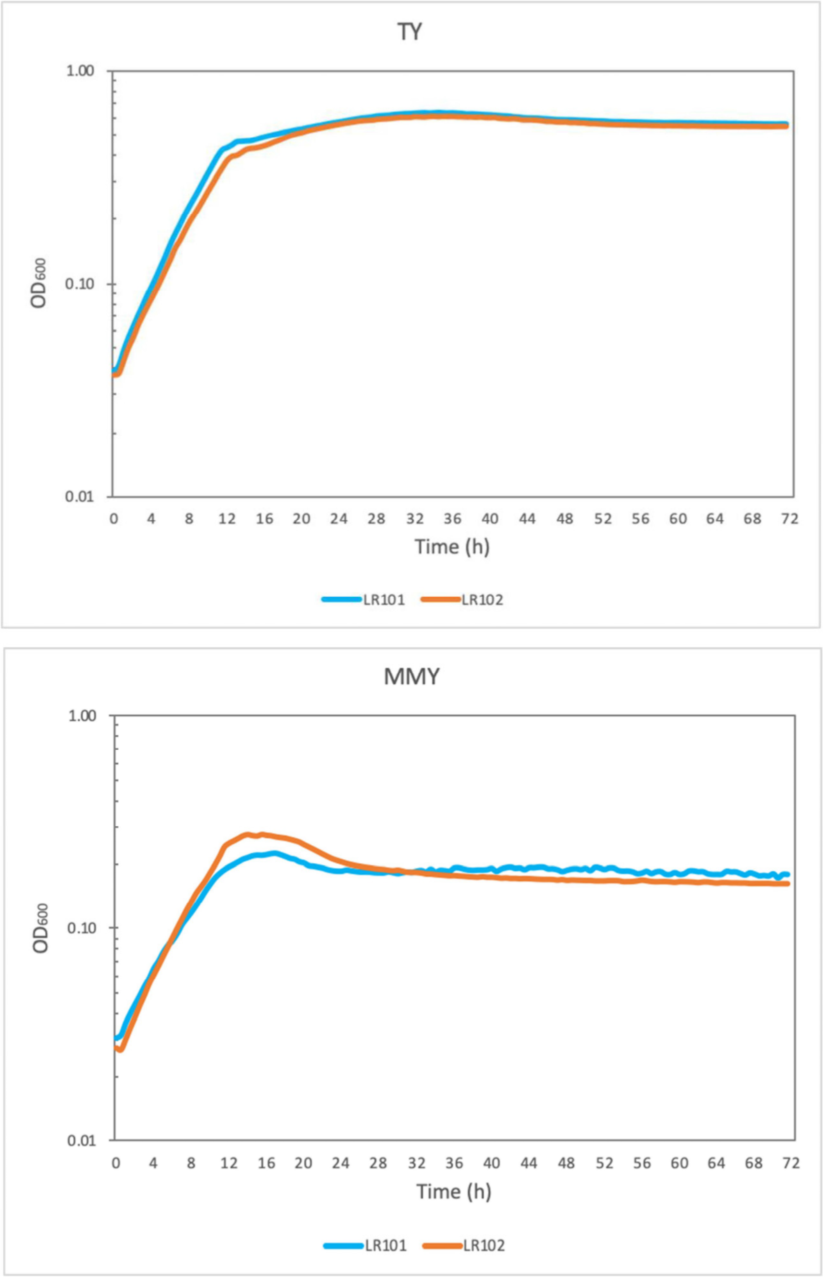

Figure S2. Growth curves of the *R. etli* strains LR101 and LR102. Rich medium TY or MMY medium with succinate as the carbon source were used. The cultures were incubated for 72h with continuous shaking at 28 °C, and OD600 was measured every 30 minutes in a Bioscreen C.
